# Circadian Temperature in Moderate to Severe Acute Stroke Patients

**DOI:** 10.5334/jcr.241

**Published:** 2024-08-01

**Authors:** Jakob Ginsbak Notland, Helle K. Iversen, Poul Jennum, Anders S. West

**Affiliations:** 1 Clinical Stroke Research Unit, Department of Neurology, Copenhagen University Hospital –Rigshospitalet, Copenhagen, Denmark; 2 Department of Clinical Medicine, University of Copenhagen, Copenhagen, Denmark; 3 Danish Center for Sleep Medicine, Department of Neurophysiology, Copenhagen University Hospital –Rigshospitalet, Copenhagen, Denmark

**Keywords:** stroke, temperature, circadian rhythm, polysomnography, neurorehabilitation

## Abstract

**Background:**

Stroke patients often present circadian disruption due to multiple causes e.g., primary disease, comorbidities, medication, immobilization, reduced daylight entrainment and sleep disturbances.

**Objective:**

To investigate the circadian rhythm of temperature in forehead skin in patients with moderate to severe stroke admitted for rehabilitation.

**Methods:**

A physiologic study in form of a secondary analysis of a former randomized study. In total 27 patients with moderate to severe stroke were included between May 1^st^ 2014, and June 1^st^ 2015. Circadian temperature was collected approx. seven days after admission at the acute stroke unit by a skin surface temperature probe as part of a Polysomnography (PSG) measurement.

**Results:**

Temperature variations show no circadian rhythm (Type 3 tests of fixed effects by SAS, *p = 0.1610*). The median temperature variance did fluctuate, but not significantly, and the small changes in circadian temperature variance did not follow the normal temperature variance.

**Conclusion:**

Patients with moderate to severe stroke show an abrogated circadian rhythm of temperature. There is an unmet need to understand the mechanisms for this, significance for stroke outcome and treatment.

## Introduction

For the human body to maintain normal physiologic function, a relatively narrow temperature frame is required, adjusting for climate factors and its own heat generation. There are two major regulatory principals involved in this process: circadian organization and homeostasis mediated through thermoregulation [1]. The natural rhythm of body temperature is a nocturnal decline facilitated by reduced heat production and vasodilatation at distal skin areas [2].

Humans maintain physiological circadian changes in body temperature as part of the circadian rhythm, controlled in the suprachiasmatic nucleus (SCN), a part of the hypothalamus [3]. This is part of the major circadian pacemaker which is entrained by exogenous cues, also called Zeitgebers where light seems to be the most important one [4].

The suprachiasmatic nucleus (SCN), also called the master clock, synchronize most circadian rhythms in the body. The ones that are easy to measure and reflect the timing of the internal temporal order are considered to be markers of the circadian rhythms. These must be periodic and recordable at frequent intervals over long periods by non-invasive methods [5]. Blood pressure, cortisol levels, heart rate, melatonin, rest/activity cycle and core body temperature have been applied as indicators of biological internal timing [6]. It takes about five days to reverse a current circadian rhythm in humans [7].

In stroke patients the circadian rhythm of melatonin is known to be disturbed which also was confirmed in this cohort [8]. Patients with moderate to severe stroke considered to be candidates for in-hospital neurorehabilitation are in high risk of developing circadian disruption because of their infarct size, related disabilities, and immobilization. This combination results in lack of natural light from the sun and many hours of artificial light from the hospital indoor lighting in evening and at night.

The skin, internal body (i.e. intestines and blood vessels), and hypothalamus contains thermal receptors that project signals via the dorsal horn and thalamus to the insular cortex, resulting in thermal sensation and cold influence thermal behavior [9]. These signals also travel to the hypothalamus where the preoptic area is considered to be the main area for thermoregulation. One should also mention the lateral hypothalamus which behavioral thermoregulation is dependent upon, and the ventromedial preoptic area which is critical to thermoregulation and the generation of fever [10].

The golden standard for measuring core body temperature has traditionally been rectal or ear temperature [11,12]. Studies have shown that forehead skin temperature and its circadian profile is proved to be closely parallel to that of rectal temperature [2,11].

A stroke compromises blood flow to the affected parts of the brain, causing cell death and surrounding edema, damaging adjacent parenchyma. Cortical strokes are shown to disturb the 24-hour circadian activity rhythm [13]. The body temperature and stroke is significantly and independently related to initial stroke severity, infarct size, mortality, and outcome [14]. However, to our knowledge, no previous studies have followed the circadian changes in temperature measured by forehead skin temperature.

In this present study of moderate to severe subacute stroke patients, we aimed to determine whether the forehead skin temperature showed a circadian rhythm and if this could present a way of measuring general disturbance of the human body circadian rhythm.

## Materials and Methods

### Study designs and participants

This study is part of a randomized study of the effects of naturalistic light at the Stroke Unit, Department of Neurology, Rigshospitalet, Denmark. Stroke patients who required more than two weeks of in-hospital neurorehabilitation were recruited from May 2014 to June 2015. Patients were excluded if they could not give informed consent because of their awareness status, severe aphasia, or if they were expected to be hospitalized in the rehabilitation unit for less than two weeks. Safety precautions were not necessary regarding assessments and interventions.

Circadian forehead skin temperature was recorded from 5 pm in the evening until approx. 7 am in the morning (14 hours), approx. seven days after admission at the acute stroke unit by a skin surface temperature probe as part of a Polysomnography (PSG) measurement (SOMNOscreenTM).

The study was approved by the Danish scientific ethics committee (H-4-2013-114) and the Danish Data Protection Agency (2007-58-0015). ClinicalTrials.gov Identifier: NCT02186392. A more detailed methods description has been published elsewhere [15].

### Statistical analysis

Due to several invalid recordings (missed recordings caused by the probe falling off or become loose), nine patients had to be omitted resulting in 27 included patients in total. The temperature was measured with an accuracy within milliseconds (epoch). Because of an epoch period of milliseconds, there were several missed data that were estimated to be related to movements and sweat, why we chose the highest recording during 2-hour intervals.

All analyses were performed using SAS (SAS Inst. Inc., Cary, NC USA, 9.4). A p value of <0.05 was considered significant. Continuous variables are presented as means and categorical variables are presented as number of cases. The temperature data were normally distributed, so data were not logarithmically transformed before analysis. A mixed-model analysis (Proc Mixed, SAS) was used to describe the median variances/changes among the seven time-points when time point seven is set to zero. Deviation is described as interval with lower and upper median values. The change in temperature is therefore in relation to time point 5 pm–7 am.

## Results

Ninety patients were included in the main study, of these 36 patients got PSG with temperature measurement, and we obtained useful temperature data from 27 patients.

The mean age of the patient group was 73.2 years and 44% were female. Table 1 describes the patient characteristics including smoking, hypertension, diabetes, atrial fibrillation, Barthel and NIHHS score.

**Table 1:** **Basic demographics**. *Hypertension defined as under medical treatment for hypertension at study inclusion.


CHARACTERISTIC	PARTICIPANTS (N = 27)

Mean age, years (range)	73.2

Gender	

*Male*	15

*Female*	12

Actual smoker	21

Hypertension*	18

Diabetes	

*Type 1*	0

*Type 2*	6

Hypercholesterolemia	5

Atrial fibrillation	4

Barthel score	56.3

NIHSS score	5.6


**Figure 1:**
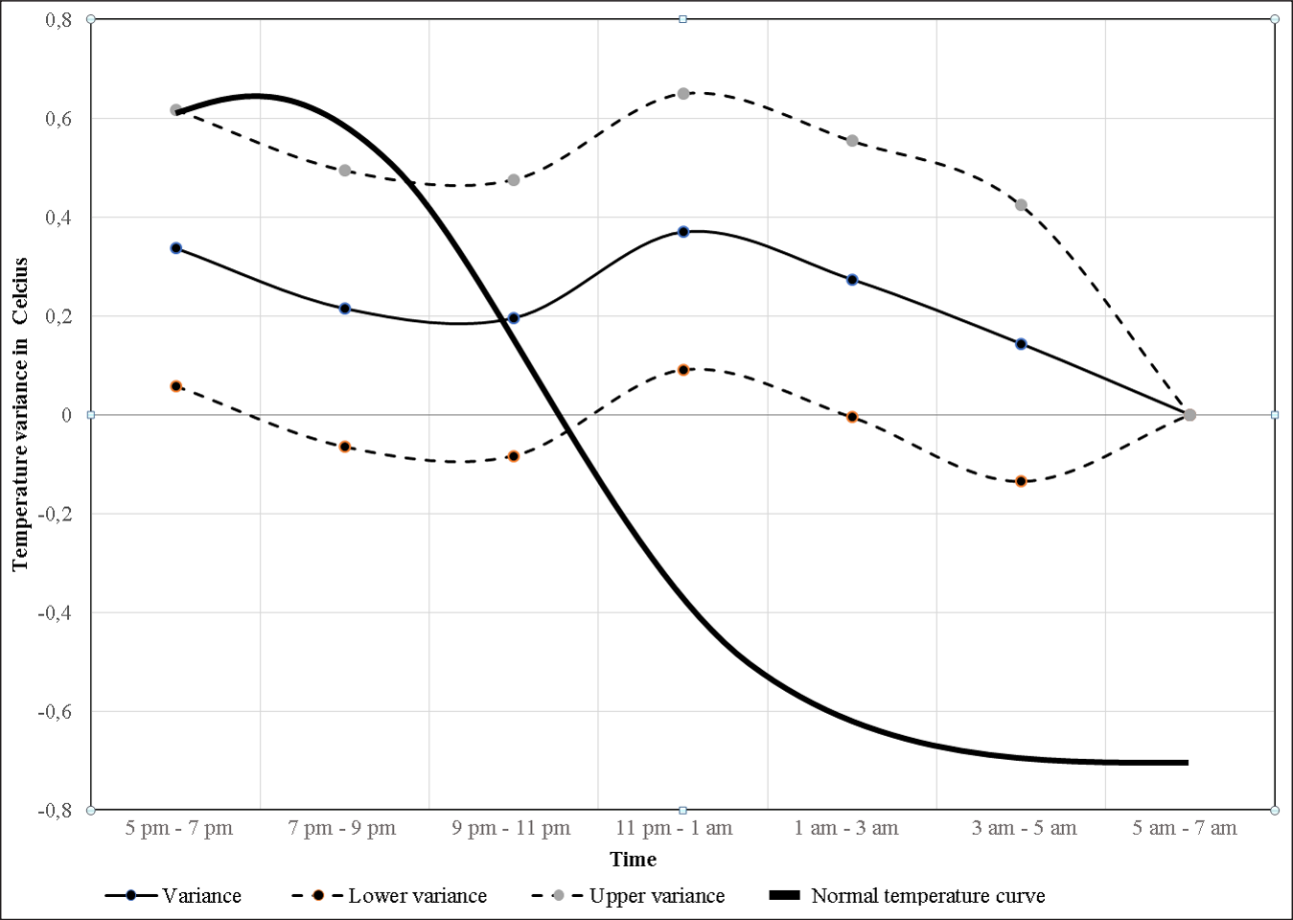
**Circadian temperature variance**. Figure 1 illustrates data from Table 2 with the measured median circadian temperature variance (narrow bold line) with lower and upper temperature reference levels (dashed lines). The thick bold line is the normal known physiological circadian core body temperature and its temperature variance during the measured time period in relation to the time-point reference 5 am–7 am. The y-axis illustrate temperature levels in Celsius and the x-axis illustrate time interval for the collected temperature period.

**Figure 2:**
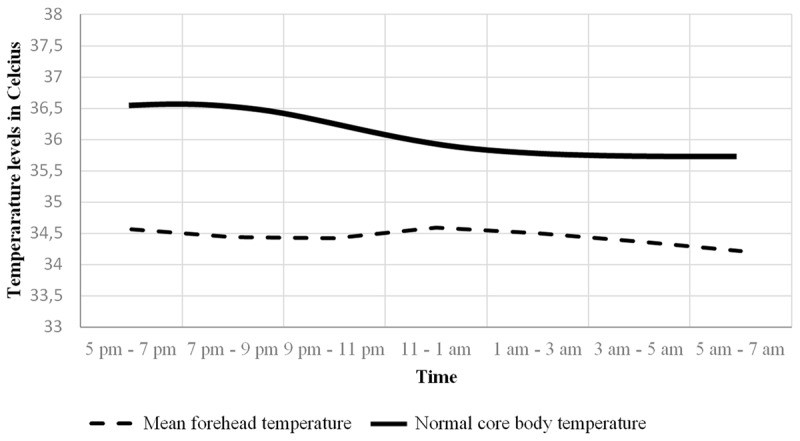
**Mean forehead temperature**. The mean forehead temperatures for each time interval are shown with a dashed line. The bold line illustrates normal known physiological core body temperature at each time interval. The y-axis illustrate temperature levels in Celsius and the x-axis illustrate time interval for the collected temperature period.

No normal temperature rhythm or significant rhythmicity in temperature were present over time, meaning there was no normal temperature circadian rhythm present in the included patients (Type 3 tests of fixed effects for rhythmicity during collected time period, p-value = 0.16) (Table 2). Figure 1 illustrates these data including lower and upper temperature variance levels, using the median temperature in the morning, between 5 am–7 am as baseline/reference. In the background is the normal temperature rhythm illustrated for comparison [16]. The tendency was a declining temperature in the evening between 5 pm and 9 pm, followed by a rise in late evening between 9 pm and 11 pm, ending with a decline during the morning between 11 pm and 7 am. Compared to the normal temperature rhythm variance, our findings show a more flattened curve. The mean temperatures at each time interval are illustrated in Figure 2. Our mean forehead temperatures are compared to normal body temperatures [16].

**Table 2:** **Variance between temperature time-points**. The variance at each time-point describes the difference in Celsius from the time-point baseline/reference “5 am–7 am”. The total circadian variance is calculated by a SAS type 3 tests of fixed effects. NS = Not significant.


TIME	MEDIAN TEMPERATURE VARIANCE IN	INTERVAL		*p* VALUE
		
	CELSIUS	LOWER	UPPER	

5 pm–7 pm	0.337	0.058	0.617	0.018

7 pm–9 pm	0.215	–0.065	0.494	NS

9 pm–11 pm	0.196	–0.083	0.476	NS

11 pm–1 am	0.370	0.091	0.650	0.010

1 am–3 am	0.274	–0.005	0.554	NS

3 am–5 am	0.144	–0.135	0.424	NS

5 am–7 am	0			

**Total circadian variance**				**NS**


## Discussion

This is the first study of circadian temperatures measured by forehead skin temperature in moderate to severe stroke patients in the subacute phase.

We demonstrated that the normal human circadian temperature rhythm in these stroke patients is disrupted, and there was no significant rhythmicity to be found compared to the normal circadian temperature.

The measured disrupted circadian temperature rhythm seems to correlate with the disturbed melatonin rhythm measured in the same stroke cohort which is published elsewhere [8]. This may demonstrate that using temperature as an indicator for general disturbance of the circadian rhythm, could substitute melatonin measuring. However, by these data it is not possible to conclude whether it is a lack of zeitgebers, especially light, or whether it is the brain damage itself that has resulted in the disturbed circadian temperature rhythm.

In addition to a disturbed temperature rhythm, we have shown a lower mean temperature than normal. The reason for this might be an older population and a disrupted autonomous function which is often seen in stroke patients [17].

Regarding continuous temperature measurements, monitoring skin temperature is considerably less invasive than measuring rectal temperature, which is in literature estimated as more precise. However, no comparative studies have been done. It is also worth to mention that rectal temperature are not without error, and an even more precise method would be measuring esophageal temperature [18]. Compared to other marker rhythms, for example melatonin, skin temperature is additionally more economic and easier accessible through numerous devices as PSG and smart watches.

This study shows that the temperature rhythm is disrupted which supports the published literature, that the circadian rhythm seems to be disturbed after stroke.

### Limitations and strengths

The main limitation of this study was the large portion of patients where the temperature data was not obtained due to electrode problems. We did not register temperature changes for 24 hours, but 14 hours, since the temperature collection was done during the duration of a PSG examination. The time frame from 5 pm to 7 pm is when there is the most change in body temperature, our data is therefore considered a close proxy for circadian rhythm. In addition to the presumed disrupted autonomous function in this cohort, may skin temperature be influenced by changes in wind, chill, radiation, movements, changes in ambient temperature, humidity and changes in local skin blood flow [19]. In contrast to finger temperatures the forehead temperature is not influenced by pain [20]. Other confounding factors can have been infections and anti-inflammatory medication, elements that was not registered.

The strength of this study was the accessibility of the data-gathering, using the temperature sensor from the PSG-device. Instead of the planned standard temperature measurements, as part of the vitals registered by the nurses, we had continuous measurements minimizing the risk of errors and false values.

## Conclusion

The aim of this study was to map circadian temperature changes in admitted acute stroke patients using forehead skin temperature measurement. We showed that there was no significant rhythmicity, probably in relation to a flattened circadian temperature which also was seen in the circadian rhythm of melatonin in this cohort. These findings may demonstrate that the thermoregulation in stroke patients is disturbed, and that skin temperature is a valuable vital parameter representing more excessive homeostatic damage than the obvious cortical signs. Forehead skin temperature may be an applicable representative for circadian disturbance and further research should address this issue.
